# Rapid Detection of Zika Virus in Urine Samples and Infected Mosquitos by Reverse Transcription-Loop-Mediated Isothermal Amplification

**DOI:** 10.1038/s41598-018-22102-5

**Published:** 2018-02-28

**Authors:** Laura E. Lamb, Sarah N. Bartolone, Maya O. Tree, Michael J. Conway, Julien Rossignol, Christopher P. Smith, Michael B. Chancellor

**Affiliations:** 1 Department of Urology, Beaumont Health System, Royal Oak, MI United States of America; 2 Oakland University William Beaumont School of Medicine, Rochester Hills, MI United States of America; 3 Foundational Sciences, Central Michigan University, College of Medicine, Mt. Pleasant, MI United States of America; 4 Field Neurosciences Laboratory for Restorative Neurology, Central Michigan University, Mt. Pleasant, MI United States of America; 5 Program in Neuroscience, Central Michigan University, Mt. Pleasant, MI United States of America; 6 Scott Department of Urology, Baylor College of Medicine, Houston, TX United States of America

## Abstract

Infection with Zika virus (ZIKV) is of growing concern since infection is associated with the development of congenital neurological disease. Quantitative reverse transcription PCR (qRT-PCR) has been the standard for ZIKV detection; however, Reverse Transcription Loop-Mediated Isothermal Amplification (RT-LAMP) may allow for faster and cheaper testing. Studies have suggested that ZIKV detection in urine is more sensitive and has a longer window of detection compared to serum and saliva. The objective of this study was to develop a urine diagnostic test that could be completed in under 30 minutes. Urine samples spiked with ZIKV or dengue virus were tested using RT-LAMP as well as by conventional quantitative qRT-PCR. These techniques were then validated using crude lysates made from ZIKV infected mosquitoes in addition to urine and serum samples from ZIKV infected patients. RT-LAMP specifically detected ZIKV in urine and serum for ZIKV infected patients and crude mosquito lysates. This test was performed in under 30 minutes and did not require RNA extraction from urine nor mosquitos. This approach could be used for monitoring of exposed individuals, especially pregnant women, couples wanting to conceive, or individuals with suspicious symptoms as well as surveillance of mosquito populations.

## Introduction

Infection with Zika virus (ZIKV) is of growing concern since infection during pregnancy can lead to miscarriage and severe birth defects including microcephaly^1^. ZIKV is spread by *Aedes aegypti* but can also be transmitted sexually^1^. There is currently no vaccine or targeted therapeutic for ZIKV.

It is difficult to diagnose ZIKV infection based on clinical symptoms alone due to overlap with other arboviruses, such as Dengue virus (DENV). In addition, infection is asymptomatic in 60–80% of adult patients^1^. Quantitative reverse transcription PCR (qRT-PCR) for ZIKV in both serum and urine within the first 14 days of infection is currently the gold-standard for diagnostic molecular testing^2^. Testing for ZIKV infection is complicated by a limited window of virus replication in infected individuals and variation in viral load depending on the sample type. For example, ZIKV can be detected for a longer timeframe and at higher expression levels in urine compared to serum and saliva, although detection in serum may occur at an earlier time point after infection^3–5^. Arbovirus surveillance in vector mosquitoes is also subject to limitations related to both sensitivity and specificity and the need for expensive equipment and trained personnel.

Reverse transcription loop-mediated isothermal amplification (RT-LAMP) is a one-step nucleic acid amplification method based on PCR technology that has been used to diagnose infectious diseases^6^. Advantages of RT-LAMP include: (1) high specificity; (2) high sensitivity; (3) short turn-around time; (4) robustness in various pH and temperature ranges^7^; (5) low cost and stability of reagents at room temperature; (6) it has been an applied technology for bacterial infection in urine^8^.

This study describes a RT-LAMP methodology that can detect ZIKV in patient samples and crude *Aedes aegypti* lysates without RNA isolation. The test could be used at the point-of-care by untrained personnel for the monitoring of exposed individuals as well as surveillance of disease vectors.

## Results

### RT-LAMP is specific and sensitive for Zika Virus

**Figure 1:**
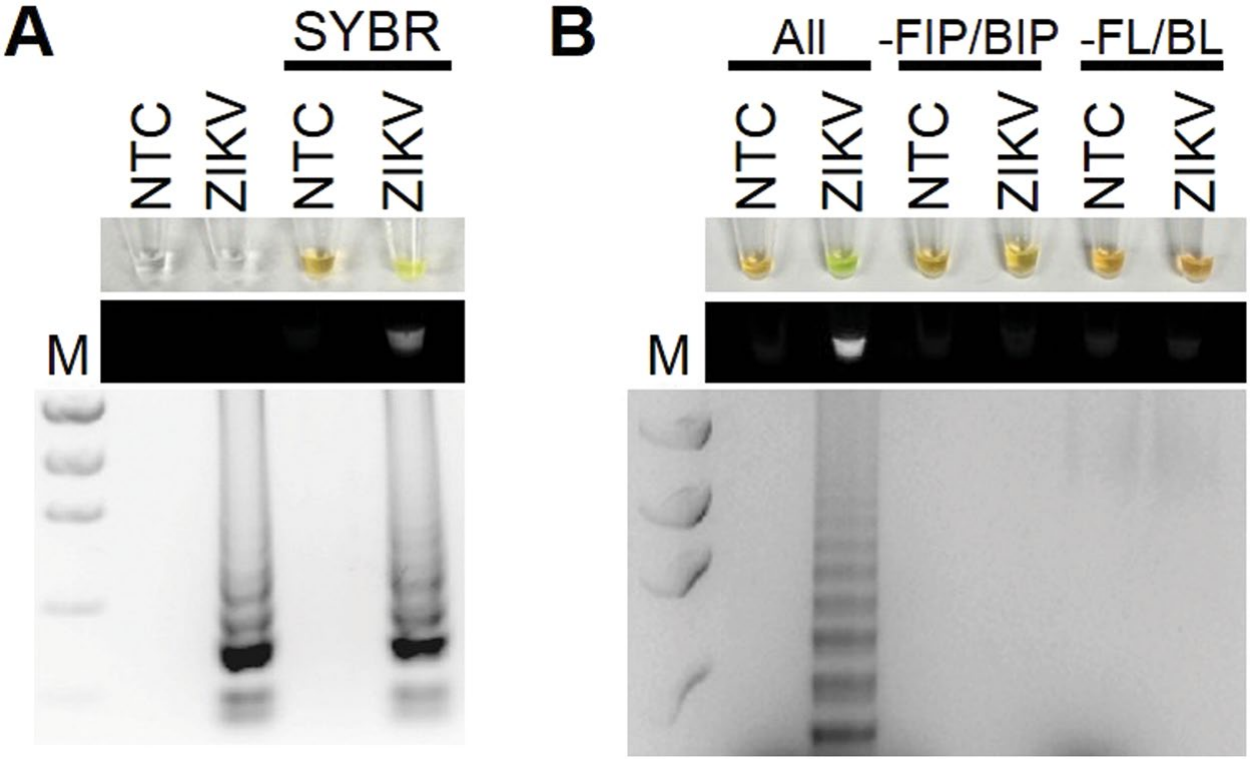
RT-LAMP detection of ZIKV. (**A**) ZIKA RT-LAMP amplification of ZIKV PCR Standard (ZIKV; Robert Koch Institute) but not no template control (NTC; negative control) as visualized by addition of SYBR Green I (SYBR) by eye (upper panel), green fluorescence (middle panel), or gel electrophoresis (bottom panel). Lane M: Low DNA Mass Marker (ThermoFisher Scientific). (**B**) All primers (All) are required for effective LAMP reaction. Reactions without FIP and BIP (-FIP/BIP) or FL and BL (-FL/BL) resulted in a negative RT-LAMP reaction.

**Figure 2:**
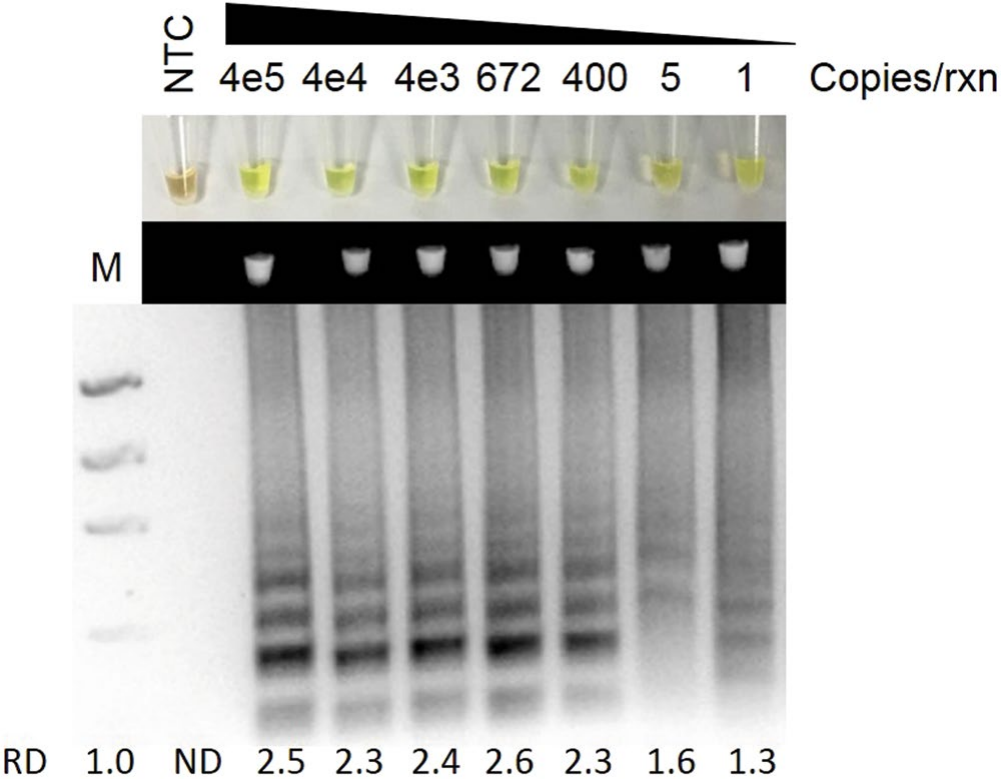
ZIKV RT-LAMP sensitivity for ZIKV. Sensitivity assessment of ZIKV RT-LAMP using serial dilutions of ZIKV PCR Standard (Robert Koch Institute) from 4 × 10^5^ genome copies/reaction to 1 copies/reaction as visualized by addition of SYBR Green I by eye (upper panel), green fluorescence (middle panel), or gel electrophoresis (bottom panel). Lane M: Low DNA Mass Marker (ThermoFisher Scientific); NTC: No template control (negative control). Relative density (RD) of the entire bands for a column relative to the DNA Mass Marker are indicated below the corresponding lane. Lanes that did not have any detectable bands over background are reported as not detectable (ND).

To establish the optimal conditions for RT-LAMP using a ZIKV PCR control, several primer sets, ranges of temperatures (57–65 °C), and incubation times (30–60 mins) were tested. The best amplification results were obtained at 61 °C for 30 mins as indicated by a banding pattern after electrophoresis on a gel (Fig. 1A). Positive reactions containing SYBR Green I could be observed by naked eye by a color change from orange to yellow, under fluorescent light in response to UV excitation, or by laddering pattern of bands after electrophoresis on a gel (Fig. 1A). The RT-LAMP reaction required all 6 primers to work under the optimized conditions; removing the forward and backward inner primers or the loop primers did not result in a positive result (Fig. 1B). In order to determine the lower detection limit of the RT-LAMP reaction for ZIKV, a dilution series ranging from 1 × 10^3^ to 1 × 10^8^ PFU ZIKV was amplified (Fig. 2). The limit of detection was approximately equivalent to 1 genome.

**Figure 3:**
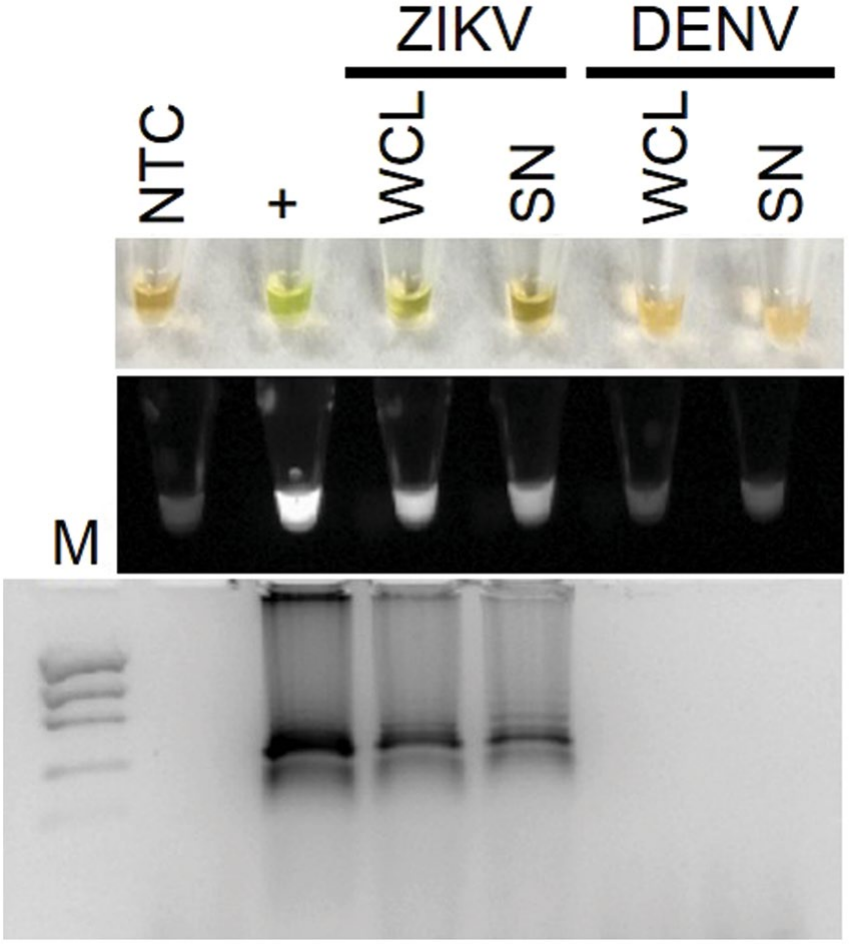
ZIKV RT-LAMP specificity for ZIKV. Specificity assessment of ZIKV RT-LAMP in ZIKV or DENV infected whole cell lysates (WCL) or cell culture supernatants (SN) as visualized by the addition of SYBR Green I by eye (upper panel), green fluorescence (middle panel), or gel electrophoresis (bottom panel). Lane M: Low DNA Mass Marker (ThermoFisher Scientific); NTC: No template control (negative control); + : ZIKV PCR Standard (Robert Koch Institute; positive control).

We then determined the specificity of the RT-LAMP assay by testing whole cell lysates and supernatant from *Aedes albopictus* C6/36 cells infected with ZIKV or DENV, a closely related flavivirus. Only whole cell lysates and supernatants from ZIKV infected cells, but not DENV, had positive RT-LAMP reactions indicating specificity of the reaction for ZIKV (Fig. 3).

### Zika Virus and 18 *S rRNA* detection in urine

**Figure 4:**
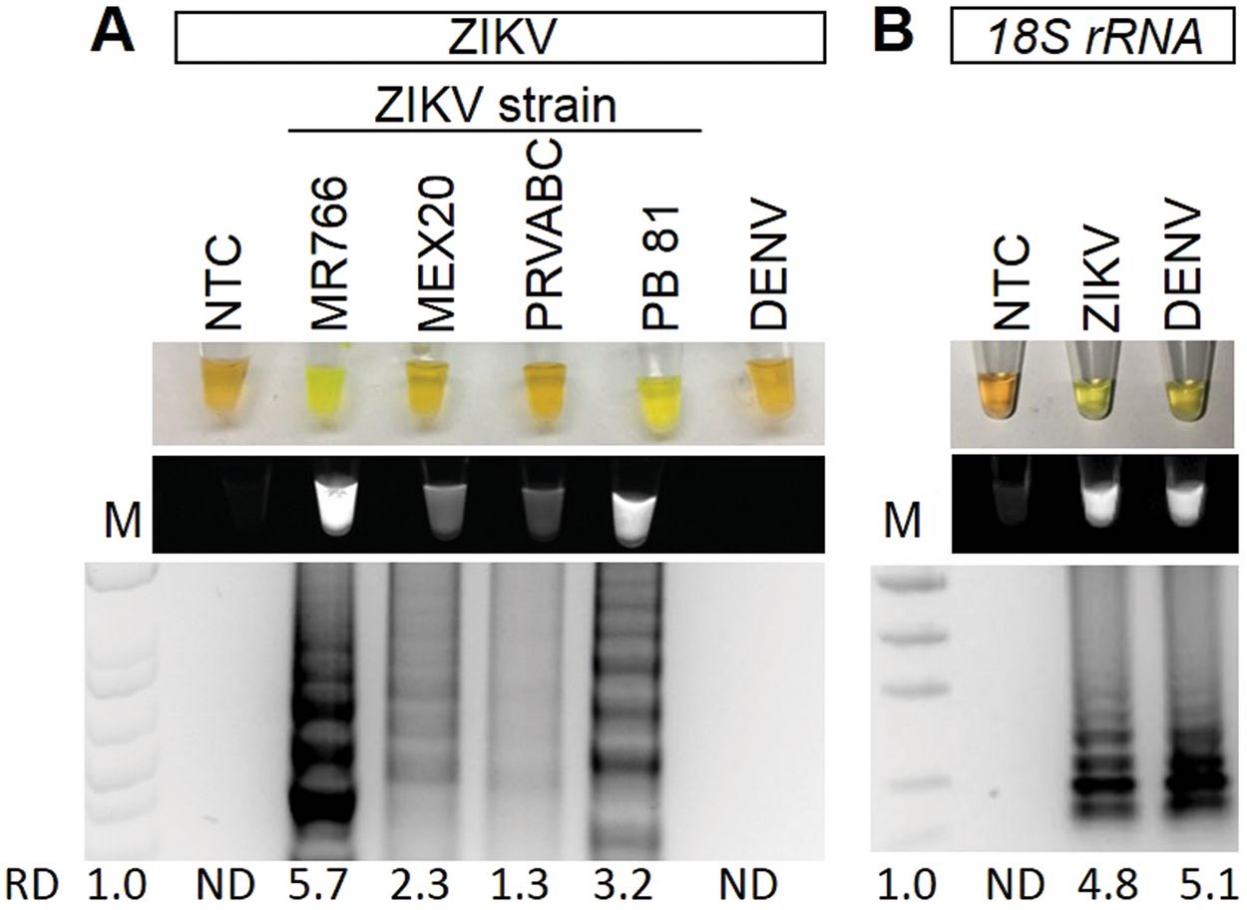
ZIKV and *18S rRNA* RT-LAMP in simulated urine samples. Urine samples were spiked with either different strains of ZIKV or DENV and subjected to a ZIKV (**A**) or *18S rRNA* (**B**) specific RT-LAMP reaction then visualized by addition of SYBR Green I by eye (upper panel), green fluorescence (middle panel), or gel electrophoresis (bottom panel). Lane M: Low DNA Mass Marker (ThermoFisher Scientific); NTC: No template control (negative control). Relative density (RD) of the entire bands for a column relative to the DNA Mass Marker are indicated below the corresponding lane. Lanes that did not have any detectable bands over background are reported as not detectable (ND).

To determine if RT-LAMP could detect ZIKV in human urine samples, human urine samples were spiked with ZIKV. To demonstrate specificity, some urine samples containing DENV were also tested. Urine samples were directly used for RT-LAMP without performing RNA isolation. RT-LAMP for ZIKV was positive for ZIKV containing urine samples for all the ZIKV strains tested to varying degrees but not for DENV positive samples, demonstrating that RT-LAMP could work in reactions containing urine and was specific (Fig. 4A). The sequence of the RT-LAMP primers were also compared to aligned sequences of related arboviruses including Chikungunya (CHIKV), DENV, Japanese encephalitis virus (JEV), West Nile virus (WNV), and Yellow Fever Virus (YFV) (Supplemental Table S1). The percent nucleotide mismatch varies from 29.6% to 52.5%, which makes it highly unlikely for there to be false positive detection with these other viruses. In order to have a quality control for RT-LAMP reactions, we designed a RT-LAMP reaction specific to human 18 S ribosomal ribonucleic acid (18 *S rRNA*). 18 *s rRNA* has good integrity in human urine and is often used for normalization of RNA^9–11^. Both ZIKV and DENV urine samples, but not the negative no template control (NTC), were positive for 18 *S rRNA* RT-LAMP reaction (Fig. 4B).

### Zika virus and *Aedes aegypti Actin* detection in mosquitos

**Figure 5:**
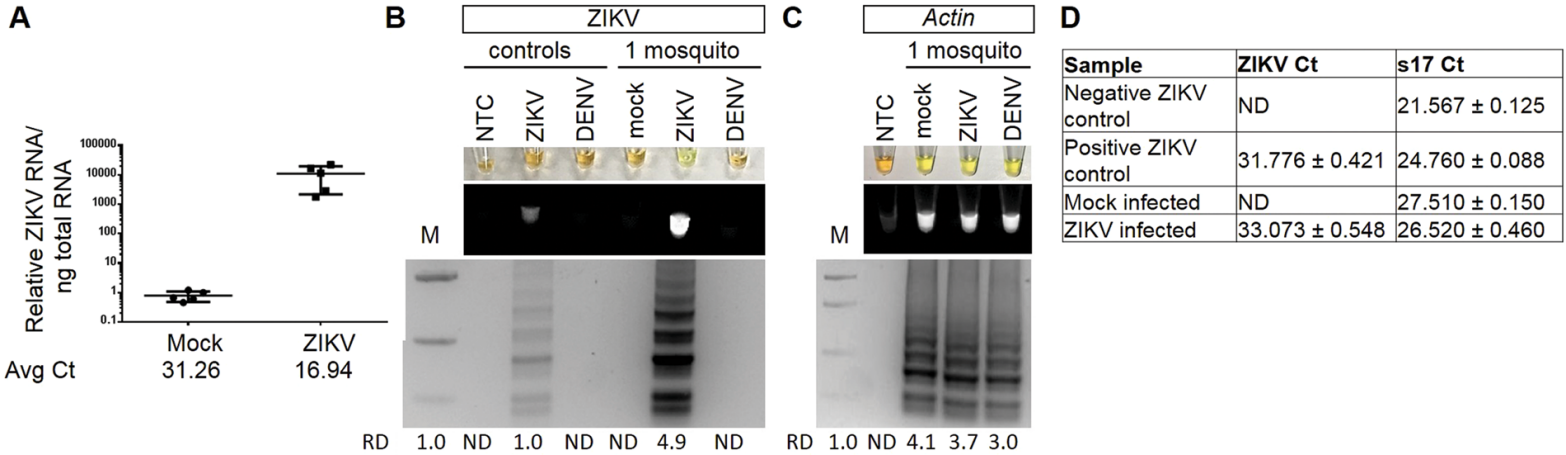
ZIKV and *Actin* detection in mosquitos. (**A**) qRT-PCR of ZIKV RNA normalized to total RNA for mock (n = 5) or ZIKV (n = 5) infected mosquitos. (**B**,**C**) Single mosquitos were infected with either mock, ZIKV, or DENV and subjected to a ZIKV (**B**) or *Aedes aegypti Actin* (**C**) specific RT-LAMP reaction then visualized by addition of SYBR Green I by eye (upper panel), green fluorescence (middle panel), or gel electrophoresis (bottom panel). Lane M: Low DNA Mass Marker (ThermoFisher Scientific); NTC: No template control (negative control). Relative density (RD) of the entire bands for a column relative to the DNA Mass Marker are indicated below the corresponding lane. Lanes that did not have any detectable bands over background are reported as not detectable (ND). (**D**) Detection of ZIKV or ribosomal s17 by qRT-PCR. Data shown as mean ± standard deviation.

We sought to determine if our ZIKV RT-LAMP was sensitive enough to detect ZIKV in a single infected mosquito. Quantitative reverse transcription PCR (qRT-PCR) was done to confirm that ZIKV was able to infect this colony of mosquitoes (Fig. 5A). Crude mosquito lysates were used for RT-LAMP without RNA isolation. In the ZIKV specific RT-LAMP reaction, there was robust detection of ZIKV from just a single ZIKV infected mosquito, but no signal from the mock or DENV infected mosquitos (Fig. 5B). Thus, the ZIKV RT-LAMP is specific and sensitive for ZIKV in as few as one single infected mosquito. To again establish a RT-LAMP reaction that could serve as a quality control, a RT-LAMP reaction was also designed against *Aedes aegypti Actin*. Actin was detected in all samples containing a mosquito but not in the no template negative control (Fig. 5C). Lastly, qRT-PCR was done using the crude mosquito lysates without RNA isolation. All crude single mosquito lysates had detectable levels of housekeeping gene s17; as expected only the positive control and ZIKV infected samples had detectable ZIKV mRNA (Fig. 5D).

### Confirmation of RT-LAMP detection of Zika virus in clinical samples

**Figure 6:**
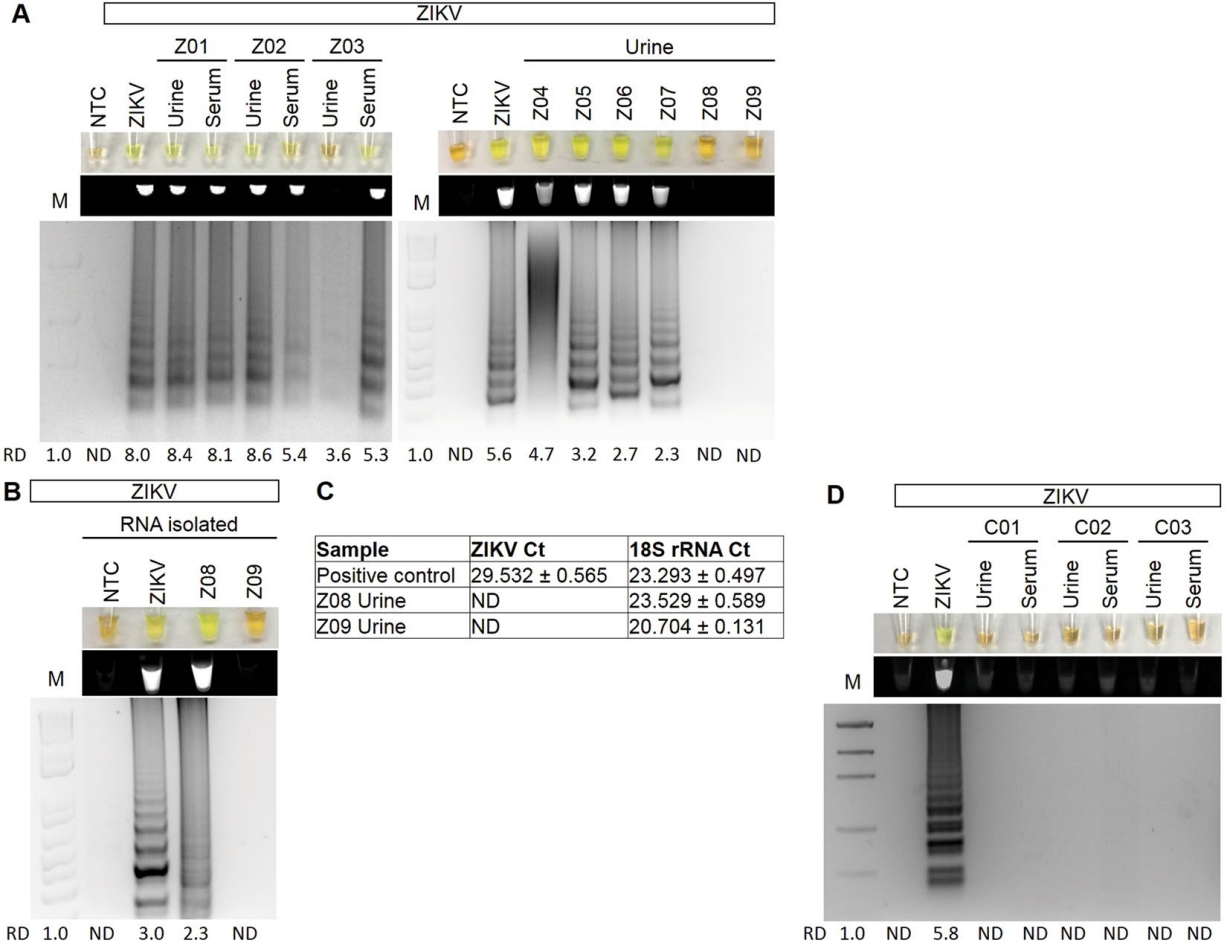
Examination of ZIKV and *18* *s rRNA* in human clinical samples. (**A**) ZIKV positive patient samples subjected to a ZIKV specific RT-LAMP reaction without (**A**) or with (**B**) RNA isolation then visualized by addition of SYBR Green I by eye (upper panel), green fluorescence (middle panel), or gel electrophoresis (bottom panel). Lane NTC: No template control (negative control); Lane ZIKV: ZIKV PCR Standard (ZIKV; Robert Koch Institute). Relative density (RD) of the entire bands for a column relative to the DNA Mass Marker are indicated below the corresponding lane. Lanes that did not have any detectable bands over background are reported as not detectable (ND). (**C**) Detection of ZIKV or ribosomal 18 S rRNA by qRT-PCR. Data shown as mean ± standard deviation. (**D**) ZIKV specific RT-LAMP in asymptomatic control patients.

To validate that the ZIKV-specific RT-LAMP could detect patients with active ZIKV infection, the RT-LAMP was performed on nine patient urine and serum samples that were collected on days 5–15 post-infection. One patient (patient #Z03, day 5 collection) had no clinical symptoms, while the other patients had mild flu-like symptoms including fever, rash, joint pain, myalgia, eye pain, cephalgia, and diarrhea. Patient #Z01 had both urine and serum samples positive for ZIKV by RT-LAMP and by IgG and IgM tests (Fig. 6A, Table 1). Patient #Z02, a women at 8 weeks pregnant, had a positive urine sample and a weakly positive serum sample by RT-LAMP (Fig. 6A). This patient also had a positive IgM and a weakly positive IgG test (Table 1). Patient #Z03, who had no symptoms, had a positive serum and negative urine for RT-LAMP. The IgM test was reported to be positive for this patient. Six other urines from patients were also tested with 4 of these being positive by RT-LAMP without RNA isolation (Fig. 6A,Table 1). Patient #Z08 was positive by RT-LAMP and not qRT-PCR, but only after RNA isolation (Fig. 6B,C, Table 1). Patient #Z09, whose urine sample was collected on day 15 post-infection, had milder symptoms than the other patients and no longer had detectable ZIKV in their urine as confirmed by qRT-PCR testing after RNA isolation (Fig. 6B,C, Table 1). Detection of 18 S rRNA by qRT-PCR demonstrated that RNA could be successfully detected in this sample. 20 asymptomatic controls from areas of low risk for ZIKV transmission were also tested with no positive results (Fig. 6B, not all data shown).

**Table 1:** Clinical samples positive for Zika Virus.

Patient^#^	Age/Gender	Symptomatic	Days Post Infection	Rapid Kit*		RT-LAMP	
				IgG	IgM	Urine	Serum
Z01	43 yo Female	Yes	6	−	+	+	+
Z02	18 yo Female	Yes	7	weak	+	+	weak
Z03	21 yo Male	No	5	n.d.	+	−	+
Z04	34 yo Female	Yes	6	n.d.	n.d.	+	n.d.
Z05	21 yo Male	Yes	7	+	+	+	n.d.
Z06	19 yo Male	Yes	9	+	+	+	n.d.
Z07	56 yo Female	Yes	13	n.d.	n.d.	+	n.d.
Z08	35 yo Female	Yes	6	n.d.	n.d.	+**	n.d.
Z09	26 yo Male	Yes	15	+	+	−***	n.d.

n.d. is not determined.

*Rapid Kit IgG and IgM testing results reported by Antibody Systems, Inc.

**Only after RNA isolation.

***Also not detectable by qRT-PCR with RNA isolation.

## Discussion

Given the rapid emergence of ZIKV and the severe birth defects that can result in children of infected mothers, improved diagnostic options that are fast, reliable, easy, and affordable are required. This is critical since ZIKV infection in adults can be asymptomatic or result in non-specific, flu-like symptoms that could also be the result of other common diseases endemic to the area, including DENV. Moreover, couples trying to conceive may not know they are infected, and as ZIKV RNA can remain detectable in semen for up to 6 months after onset of symptoms^3,12^, there is risk of unknowingly passing on ZIKV infection *in utero*. Conventional qRT-PCR, while specific and sensitive, must be done by trained personnel on specialized equipment at a qualified laboratory. This study demonstrated that RT-LAMP allows rapid detection of ZIKV in clinical urine and serum samples as well as mosquito samples. Furthermore, we have developed two robust RT-LAMP assays that can serve as a sample quality control for the RT-LAMP reactions by amplifying housekeeping genes- 18 *S rRNA* in human samples and Actin in *Aedes aegypti* mosquito samples.

Currently, clinical testing for ZIKV in the United States is done by central testing laboratories, resulting in a 2–4 week turn-around to patients for results. This study sought to improve upon this by developing a potential point-of-care test. Point-of-care testing has several advantages for emerging infectious diseases like ZIKV and must be easy to use and analyze, low cost, fast, and require little if any laboratory infrastructure without compromising on sensitivity or specificity. RT-LAMP, as a nucleic acid based test, meets these requirements and therefore has large value for screening and testing for ZIKV and other infectious diseases in potentially exposed populations, both human and endogenous vectors including *Aedes aegypti*. Only a few kits are commercially available for mosquito testing for DENV, and those are antigen based which may lack the specificity a nucleic acid test could provide. There is currently no commercially available test for ZIKV surveillance in mosquitos.

In some of the experiments in this paper, urine preservative (Norgen Biotech) was added to urine samples immediately after collection. This was done as some urine samples were collected where refrigeration was not immediately possible; therefore, preservative was required to stabilize any RNA in the samples. The presence of urine preservative in samples did not have any noticeable effect on RT-LAMP reactions compared to samples with no urine preservative. Furthermore, we found that adding urine at more than 20% of the total volume of the RT-LAMP reaction was inhibitory. An unexpected benefit was that we did not have to isolate RNA from the samples; unprocessed samples were directly used in the RT-LAMP assays. From the clinical samples tested, 7 of 8 symptomatic patients were positive by RT-LAMP and this was verified in some patients by a positive IgG or IgM tests. In cases where both urine and serum samples were tested, the urine samples gave stronger RT-LAMP signals than the serum samples. One patient, who was asymptomatic, was positive by RT-LAMP in the serum sample only. Samples of one patient first required RNA isolation for ZIKV to be detectable by RT-LAMP. However, qRT-PCR was still not able to detect ZIKV from this isolated RNA sample, suggesting that RT-LAMP may be more sensitive. One patient did not test positive for RT-LAMP, however ZIKV was also not detectable in their urine by qRT-PCR even after RNA isolation and purification, suggesting that ZIKV was no longer detectable in the urine. Although urine has a longer detection window for ZIKV than serum or saliva, it does decrease with time and this urine sample was collected 15 days after symptom onset^3^. Lastly, all 20 asymptomatic control patients, taken from geographical regions with low ZIKV infection risk, were negative for the ZIKV specific RT-LAMP reaction.

Primers were designed for a highly conserved sequence of the nonstructural protein 5 of ZIKV that was found in 16 strains and were verified in 5 different ZIKV strains and in three sample types (human urine and serum, and mosquitos). We tested a range of temperatures from 57 °C to 65 °C; all the temperatures gave comparable results thereby this particular RT-LAMP reaction had a large temperature range. We also tested a range of times from 5–60 minutes. The optimal time for detection of RT-LAMP products by color change alone was 30 minutes, however we could detect by UV light excitation or banding patterns on gels in a total incubation time of as little as 8 minutes. The addition of DMSO in our reaction completely inhibited the RT-LAMP reaction. Lastly, the use of *Bst* polymerase 2.0 WarmStart polymerase was used as this has been reported to obtain amplification signals quicker and have increased stability at room temperature compared to wild-type *Bst* DNA polymerase^13,14^.

Other groups have also developed new technologies for detection of ZIKV^15–21^, however many technologies have only been demonstrated in spiked samples, require RNA isolation and purification, have not been demonstrated to work in mosquitos, or have not been demonstrated to work in urine samples which may contain inhibitors for the proposed technology. None are yet commercially available. In contrast, we demonstrated that our diagnostic test works in both spiked and endogenously infected samples, in both human and mosquito samples, and works in both urine and serum samples. The limit of detection was approximately equivalent to 1 genome. Typical viremia of a symptomatic ZIKV infected patient is 103–106 PFU/mL^22^. Importantly, this method was able to detect ZIKV in samples without first doing a RNA isolation and purification, which significantly decreases the time and cost of this assay.

Although RT-LAMP reactions are highly specific, there are several limitations including that it is not a quantitative test. There can be a higher rate of false positives, however we did not experience this in any of our no template negative control reactions, known ZIKV negative control samples, DENV positive controls, or mock mosquito samples. The primers selected also have poor nucleotide alignment with other flaviviruses, making it highly improbable to detect these other viruses. We also took the extra precautions of having a lateral work flow for all experiments including analysis and we included Antarctic Thermolabile UDG (Uracil- DNA Glycosylase; NEB) in all reactions. UDG removes the uracil-base from DNA, thereby preventing possible carry-over contamination from previous reactions. Lastly, this study was not powered to determine sensitivity in a clinical population.

## Conclusions

A fast and robust assay was developed for detection of ZIKV specifically in urine, serum, or mosquito samples. This simple assay could be used in the field by individuals without specialty training and may provide a new diagnostic strategy for combatting Zika and other mosquito born viruses.

## Methods

### Virus strain details

ZIKV PCR-standard (strain MR766) consisted of virus stocks made from Vero E6 cell supernatants (Livia Schrick, Robert Koch Institute). African lineage ZIKV strain IB H was obtained from ATCC. DENV2 New Guinea C strain (DENV2 NGC) was obtained from the Connecticut Agricultural Experiment Station. Mexican strain ZK-HU 0165 P (MEX20), Puerto Rican strain PRVABC 59, and Brazilian strain PB 81 (H815744) were obtained from University of Texas Medical Branch (UTMB). All samples and infectious material were handled according to Biosafety level 2 standards.

### Patient samples

All studies were approved by Beaumont Health System’s Institutional Review Board (IRB). All experiments were performed in accordance with relevant guidelines and regulations. Midstream urine samples were collected by the patient and had preservative (Norgen Biotek) added immediately after collection then shipped to Beaumont and stored at room temperature. Some urine samples did not contain the preservative; after collection they were immediately centrifuged for 10 minutes at 700 g and the supernatant frozen at −80 °C until analysis. Deidentified samples were shipped to Beaumont, Royal Oak, Michigan, USA from Clinica La Esperanza, Copán Ruinas, Hondoras (IRB approval #2016-044). All study participants gave their informed consent to participate. For asymptomatic control samples collected at Beaumont Hospital in Royal Oak, Michigan (IRB approval #2014-281), written consent was obtained from all participants prior to inclusion in the study. Deidentified and confirmed ZIKV positive urine and serum samples were also obtained from Antibody Systems, Inc. which also reported Rapid Kit IgG and IgM (Lumiquick) test results to confirm ZIKV infection. Urine samples from multiple patients were tested for each assay. For some experiments, 2 µL of 2 × 10^7^ plaque-forming units (PFU) of either ZIKV MR766 or DENV2 NGC were spiked to urine samples for test validation. Water was used as a no template control.

### Cell culture

*Aedes albopictus* C6/36 cells were obtained from Erol Fikrig (Yale University School of Medicine) and were used to generate ZIKV IB H and DENV2 viral stocks. Virus was passaged in C6/36 cells and stocks generated 8 days post-infection by harvesting cell-free supernatants. C6/36 cells were maintained in DMEM containing 10% fetal bovine serum, tryptose phosphate, and antibiotics at 28 °C and 5% CO_2_.

### Mosquito experiments

*Aedes aegypti* were maintained on raisins at 27 °C and 80% humidity according to standard rearing procedures. Mosquitos were propagated by feeding defibrinated sheep blood using a Hemotek artificial membrane feeder^23^. Female mosquitoes were infected by intrathoracic microinjection and inoculated with approximately 10^3^ genome equivalents of virus in a volume of 200nL. Whole mosquitoes were harvested 5 days post-infection by placing individual mosquitoes in 100 μL phosphate-buffered saline (PBS) and storing samples at −80 °C. Prior to RT-LAMP, thawed mosquito samples were homogenized 10 times with a P10 pipet tip in the PBS and the 2 µL crude lysate was used in RT-LAMP reactions.

### RT-LAMP primer design

The consensus sequences of 16 ZIKV strains (from PubMed: NC_012532, AY632535, EU545988, KF383119, KU321639, KU497555, KU501215, KU509998, KU527068, KU707826, KU681081, KU744693, KM078957, KU179098, KM078976, KU232301, KU556802) was established when aligned with Lasergene MegAlign (DNASTAR). 18 S ribosomal ribonucleic acid (18 *S rRNA*) primers, a control for human samples, were designed to sequence NC_018932. *Ae*. *aegypti* actin primers, a control for mosquito lysates, were designed to GenBank sequence U20287. RT-LAMP primers were designed using Primer Explorer v4 (Eiken), and blasted using Primer-BLAST (NCBI) against genomes of interest. Primers selection was prioritized as described in “A Guide to LAMP primer designing” (https://primerexplorer.jp/e/v4_manual/). In addition, primers were selected to not have four guanines in a row. A set of six olgionucleotides primers were used for the ZIKV RT-LAMP assay targeting the consensus sequence of non-structural protein 5 (NS5). RT-LAMP primers are listed in Table 2. Primers sets include an outer forward primer (F3), outer backward primer (B3), forward inner primer (FIP), backward inner primer (BIP), loop forward primer (LF), and loop backward primer (LB). Primers were ordered from Integrated DNA Technologies and the FIPs and BIPs are HPLC purified.

**Table 2:** RT-LAMP Primer Sequences used to detect ZIKV, human 18 s rRNA, and *Ae*. *aegypti actin*.

Target	Primer	Sequence (5′-3′)
*Homo sapien* 18 *S rRNA*	18 SrRNA-L3	GTTCAAAGCAGGCCCGAG
	18 SrRNA-B3	CCTCCGACTTTCGTTCTTGA
	18SrRNA-FIP	TGGCCTCAGTTCCGAAAACCAACCTGGATACCGCAGCTAGG
	18SrRNA-BIP	GGCATTCGTATTGCGCCGCTGGCAAATGCTTTCGCTCTG
	18SrRNA-LF	AGAACCGCGGTCCTATTCCATTATT
	18SrRNA-LB	ATTCCTTGGACCGGCGCAAG
ZIKV	Zika-L3	GCAGAGCAATGGATGGGATA
	Zika-B3	CCCATCCTTGAGGTACAGCT
	Zika-FIP	AACCTGAGGGCATGTGCAAACCGCGGTCAGTGGAGATGACT
	Zika-BIP	CACAGGAGTGGAAACCCTCGACTGAAGTGGTGGGAGCAGAA
	Zika-LF	TCGATTGGCTTCACAACGC
	Zika-LB	GGAGCAATTGGGAAGAAGTCC
*Ae*. *aegypti actin*	Ae21-F3	CGGCGCCACCACAAGA
	Ae21-B3	TCGTGCCTGTGTTTGTCG
	Ae21-LF	ACCGCAAGGCCAAGAACCG
	Ae21-FIP	TGCTTGGTCCCTGCCTGGGAGACAGCCCACCAGAACGA
	Ae21-BIP	GAGACGAGAACGGCCCAGCGGGTCTGGTGTGTGTCTTTG

### RT-LAMP

All set-up and execution of RT-LAMP reactions were done in an enclosed room using designated pipettes and filter tips. Analysis and imaging took place in separate rooms to prevent contamination. RT-LAMP reactions were executed in a total volume of 25 μL of 1x isothermic amplification buffer, 1.4 mM dNTPs, 8 mM MgSO_4_, 1.6 μM FIP/BIP, 0.2 μM F3/B3, 0.4 μM FL/BL primers, 0.32 U/μL Bst 2.0, 1 U/μL Antarctic Thermolabile UDG, and 0.6 U/μL *WarmStart Reverse Transcriptase* in ddH_2_0. Addition of the uracil-DNA glycosylase (UDG) reduced crossover contamination from previous reactions. Reactions were set-up on ice, incubated at 61 °C for 30 minutes (10–60 minutes have been tested), and then inactivated at 80 °C for 10 minutes. In order to optimize visualization of positive reactions, 4ul SYBR Green I was added to reactions after amplification at a 1:10 dilution in TAE. All experiments have been replicated 3–10 times. Relative densities (RD) of the entire gel banding pattern relative to the DNA ladder were calculated using ImageJ software using the Gel Analysis method^24,25^. Since LAMP results in a banding pattern, the entire lane was analyzed then compared to the known concentration of DNA in the DNA Mass Marker ladder (ThermoFisher Scientific). Lanes that did not have any detectable bands over background were reported as not detectable (ND). This is a relative measurement of the size and density of the bands per image and are not for comparing across different gels.

### qRT-PCR

RNA was extracted from mosquitoes 5 days post-infection using a QIAshredder kit to homogenize tissues and a RNeasy kit to extract RNA (Qiagen). For analysis of viral RNA in mosquitoes by qRT-PCR, qRT-PCR were performed in the same closed tube with 100ng of total RNA per reaction using the Quantitect RT-PCR Kit (Qiagen) on an Eco Real-Time PCR System (Illumina) or a QuantStudio 3 (ThermoFisher) with a total reaction volume of 10 μL. For patients Z08 and Z09, RNA from 500 µL of urine was isolated with ZR Urine RNA Isolation kit (Zymo) following manufacturer’s protocol. ZIKV primers used were F 5′-AATGGGAAGGAAAGAAGAGG-3′ and R 5′-GCTGGGGTGATGAGAGTTGT-3′. Ribosomal protein s17 primers used were F 5′-CGGAGACCAAGGAGATGTTG-3′ and R 5′-CTGTAGCCCTGTGCCGATG-3′. Cycling conditions were 50 °C for 30 min and 95 °C for 15 min, followed by 45 cycles of 94 °C for 15 s, 55 °C for 30 s and 72 °C for 30 s. Relative quantities of target cDNA were determined using the Pfaffl method and a single data point from the control group was set to 1.0^26^. Ct values were reported as averages ± standard deviation.

### Analysis of RT-LAMP

RT-LAMP reactions were divided in half. One half of the RT-LAMP reaction was electrophoresed along with Invitrogen Low DNA Mass Ladder on a 1 × Nancy-520, 2% agarose gel in 1 × TAE buffer (40 mM Tris, 20 mM acetic acid, 1 mM EDTA) at 90 V for 90–120 minutes. Gels were imaged under UV light using the BIO-RAD ChemiDoc XRS + Imaging System. Lanes containing a laddered banding pattern were qualified as a positive amplification.

In addition, a 1:10 SYBR green I (Life Technologies) dilution was made in TAE buffer, then 2.0 μL of the SYBR dilution was added to the remaining half of the reaction. The subsequent visual change of color (orange to yellow) was then also used to identify positive amplifications. The SYBR green I PCR tubes were also imaged under UV light in the BIO-RAD ChemiDoc XRS + Imaging System as the reaction creates a fluorescent output.

### Data availability

All relevant data are within the paper.

## Electronic supplementary material

Supplemental Information
