# Supplementary material for: Rapid Detection of Zika Virus in Urine Samples and Infected Mosquitos by Reverse Transcription-Loop-Mediated Isothermal Amplification
Source: Sci Rep. 2018 Feb 28;8:3803. doi: 10.1038/s41598-018-22102-5 (PMC5830622; doi:10.1038/s41598-018-22102-5)
Supplement: Supplementary file 1 — Supplemental Information [file 41598_2018_22102_MOESM1_ESM.docx]

**Supplementary Information**

**Rapid Detection of Zika Virus in Urine Samples and Infected Mosquitos by Reverse Transcription-Loop-Mediated Isothermal Amplification**

Laura E. Lamb^1,2*^, Sarah N. Bartolone^1^, Maya O. Tree^3^, Michael J. Conway^3^, Julien Rossignol^3,4,5^, Christopher P. Smith^6^, Michael B. Chancellor^1,2^

^1^ Department of Urology, Beaumont Health System, Royal Oak, MI, United States of America

^2^ Oakland University William Beaumont School of Medicine, Rochester Hills, MI, United States of America

^3^ Foundational Sciences, Central Michigan University, College of Medicine, Mt. Pleasant, MI, United States of America

^4^ Field Neurosciences Laboratory for Restorative Neurology, Central Michigan University, Mt. Pleasant, MI, United States of America

^5^ Program in Neuroscience, Central Michigan University, Mt. Pleasant, MI, United States of America

^6^ Scott Department of Urology, Baylor College of Medicine, Houston, TX, United States of America

*Corresponding author: Laura E. Lamb, PhD [laura.lamb@beaumont.org](mailto:laura.lamb@beaumont.org)

**Supplemental Table S1. RT-LAMP primers alignment with other arboviruses**

| **Virus** | **GenBank** | **% Nucelotide Mismatch** |
| --- | --- | --- |
| CHIKV | AF369024 | 52.5 |
| DENV1 | NC_001477 | 29.6 |
| DENV2 | NC_001474 | 36.4 |
| DENV3 | NC_001475 | 31.5 |
| DENV4 | NC_002640 | 32.1 |
| JEV | AY303791.1 | 34.6 |
| WNV | AY646354.1 | 32.7 |
| YFV | U17066.1 | 34.0 |

**Full-length images of gels presented in Fig 1**

**
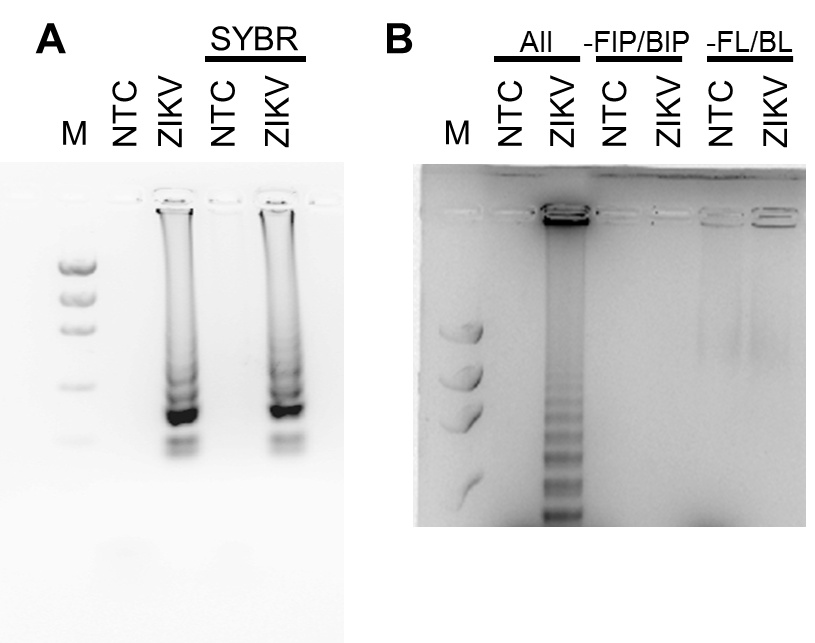
**

**Full-length images of gels presented in Fig 2**

**
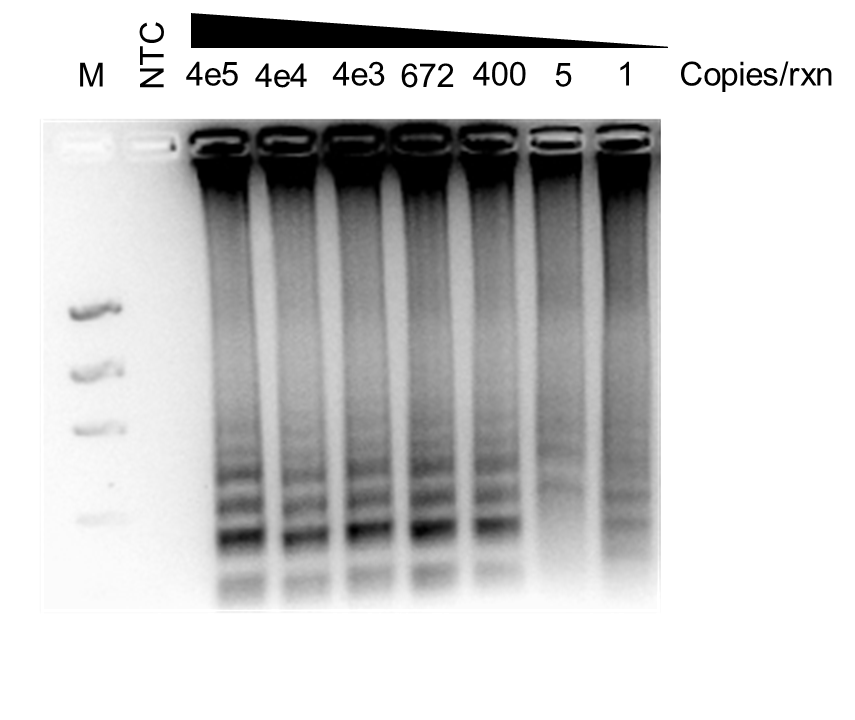
**

**Full-length images of gels presented in Fig 3**

**
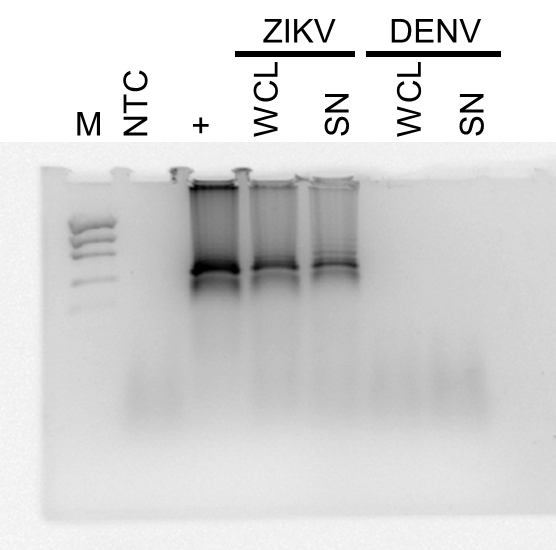
**

**Full-length images of gels presented in Fig 4**

**
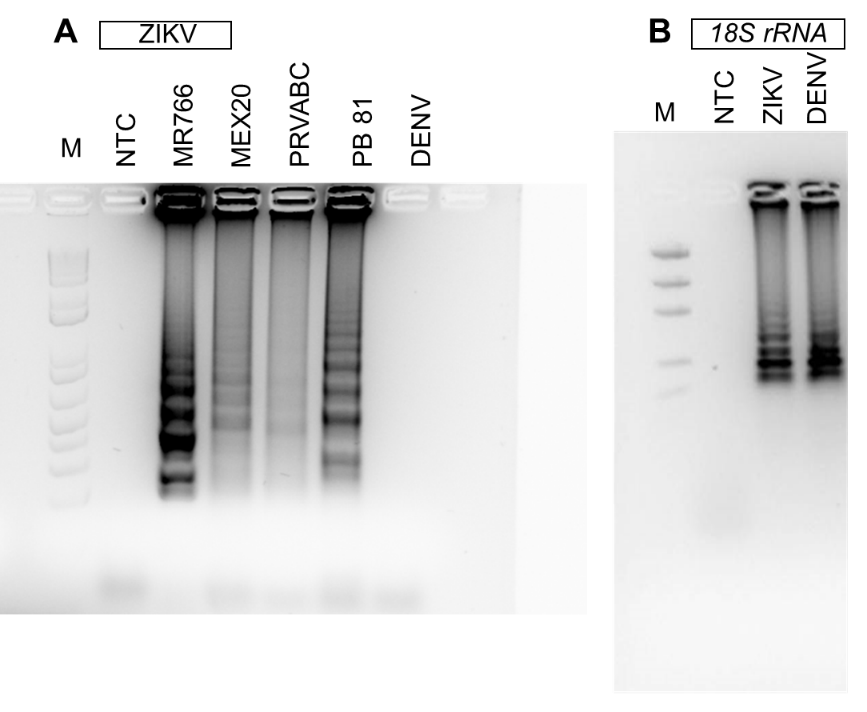
**

**Full-length images of gels presented in Fig 5**

**
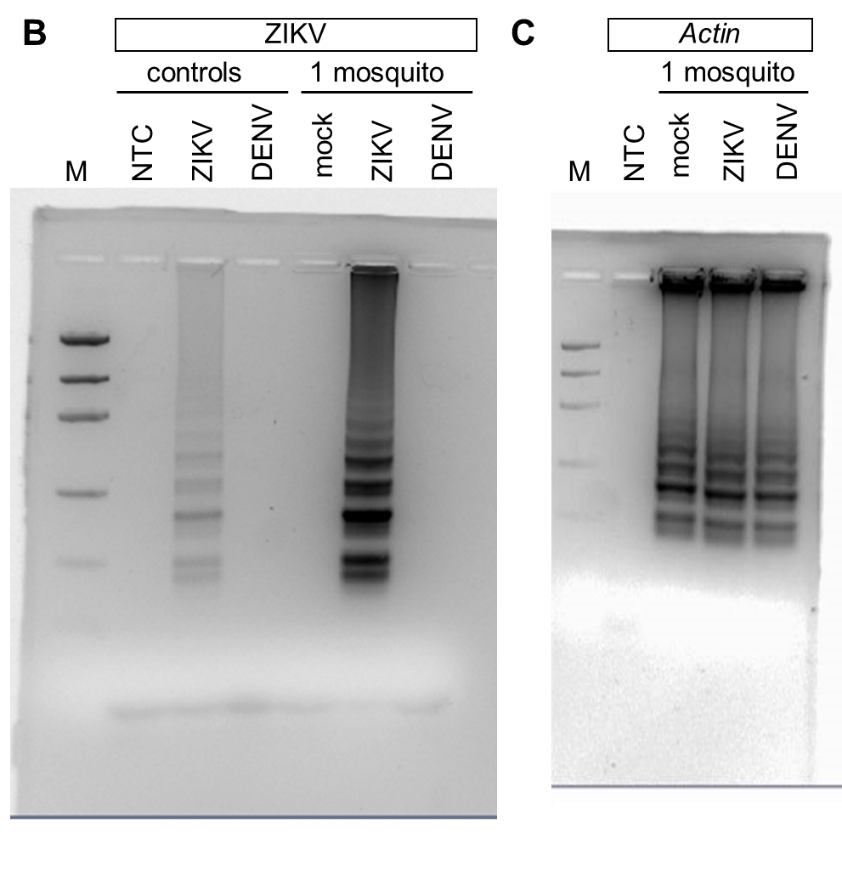
**

**Full-length images of gels presented in Fig 6**

**
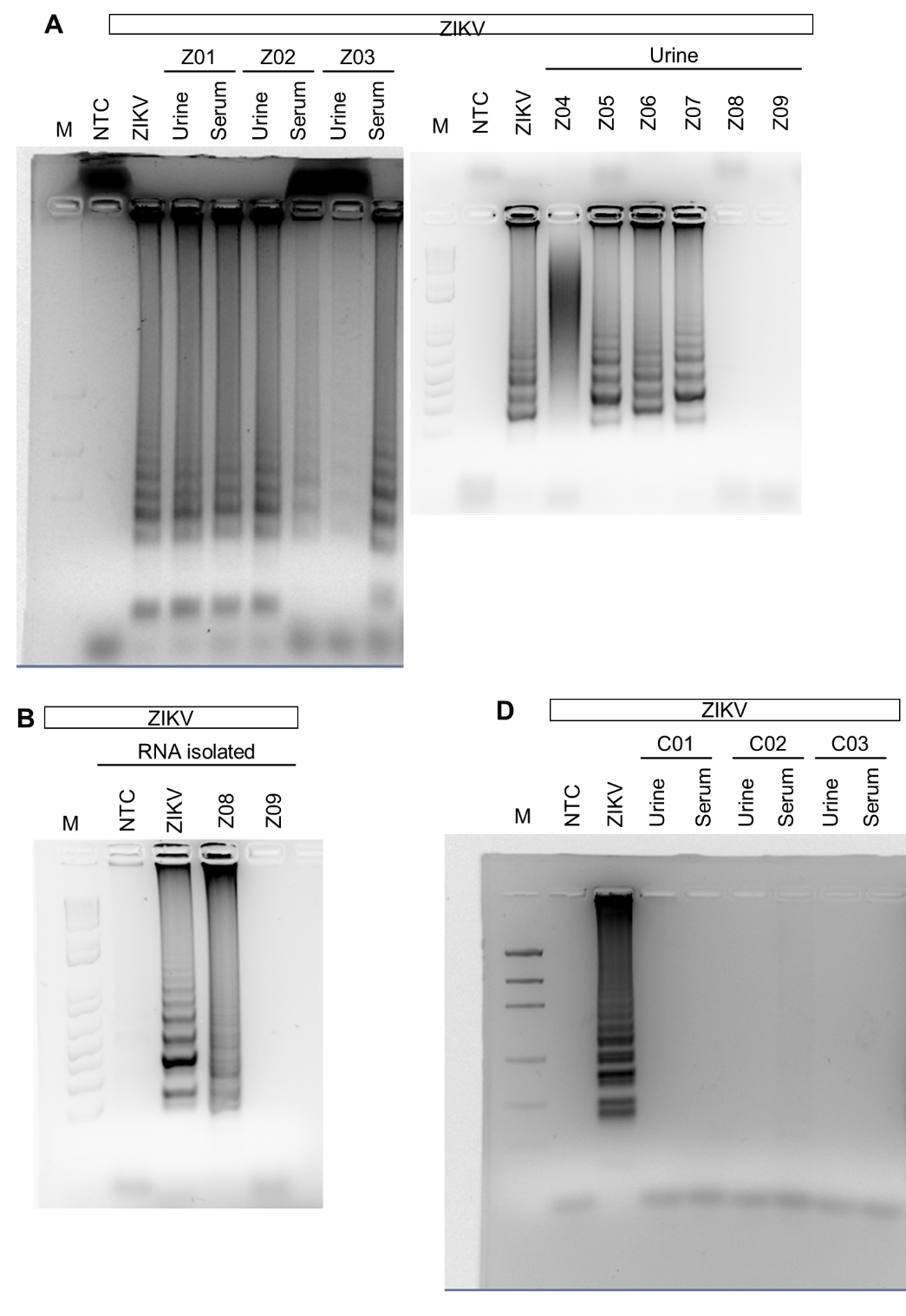
**
